# Non-randomised patients in a cholecystectomy trial: characteristics, procedures, and outcomes

**DOI:** 10.1186/1471-2482-6-17

**Published:** 2006-12-26

**Authors:** Axel Ros, Per Carlsson, Mikael Rahmqvist, Karin Bäckman, Erik Nilsson

**Affiliations:** 1 Department of Surgery, County Hospital of Ryhov, Jönköping, Sweden; 2 Center for Medical Technology Assessment, Linköping University, Linköping, Sweden; 3 Department of Surgery, University Hospital of Umeå, Sweden

## Abstract

**Background:**

Laparoscopic cholecystectomy is now considered the first option for gallbladder surgery. However, 20% to 30% of cholecystectomies are completed as open operations often on elderly and fragile patients. The external validity of randomised trials comparing mini-laparotomy cholecystectomy and laparoscopic cholecystectomy has not been studied. The aim of this study is to analyse characteristics, procedures, and outcomes for all patients who underwent cholecystectomy without being included in such a trial.

**Methods:**

Characteristics (age, sex, co-morbidity, and ASA-score), operation time, hospital stay, and mortality were compared for patients who underwent cholecystectomy outside and within a randomised controlled trial comparing mini-laparotomy and laparoscopic cholecystectomy.

**Results:**

During the inclusion period 1719 patients underwent cholecystectomy. 726 patients were randomised and 724 of them completed the trial; 993 patients underwent cholecystectomy outside the trial. The non-randomised patients were older – and had more complications from gallstone disease, higher co-morbidity, and higher ASA – score when compared with trial patients. They were also more likely to undergo acute surgery and they had a longer postoperative hospital stay, with a median 3 versus 2 days (p < 0.001 for all comparisons). Standardised mortality ratio within 90 days of operation was 3.42 (mean) (95% CI 2.17 to 5.13) for non-randomised patients and 1.61 (mean) (95%CI 0.02 to 3.46) for trial patients. For non-randomised patients, operation time did not differ significantly between mini-laparotomy and open cholecystectomy in multivariate analysis. However, the operation for laparoscopic cholecystectomy lasted 20 minutes longer than open cholecystectomy. Hospital stay was significantly shorter for both mini-laparotomy and laparoscopic cholecystectomy compared to open cholecystectomy.

**Conclusion:**

Non-randomised patients were older and more sick than trial patients. The assignment of healthier patients to trials comparing mini-laparotomy cholecystectomy and laparoscopic cholecystectomy limits the external validity of conclusions reached in such trials.

## Background

External validity of conclusions reached in a randomised trial depends upon the number and characteristics of eligible patients not included in the trial (excluded by patient or researcher) and number and characteristics of patients who do not meet the eligibility criteria [1]. According to the CONSORT [2] recommendations, the external validity of a study should be reported. However, in surgical trials, there is often a lack of data concerning the pre-randomisation collection of patients.

The laparoscopic technique has been adopted as the method of choice for cholecystectomy, and epidemiological studies indicate that 70% to 80% of all cholecystectomies are now completed laparoscopically [3-5]. However, in single-blind, randomised controlled trials the difference in convalescence between small-incision open surgery (mini-laparotomy cholecystectomy) and laparoscopic cholecystectomy is small and of short duration [6,7]. No previous study has reported data on non-randomised patients. The present report describes characteristics, operation time, postoperative hospital stay, and postoperative mortality of all non-randomised cholecystectomy patients not included in a randomised controlled trial [7].

## Methods

### Patients

Data for all patients over age 18 advised to undergo elective or acute cholecystectomy were prospectively recorded at four non-university hospitals and one university centre. Patients undergoing cholecystectomy as adjunct to other major abdominal operations such as pancreatic resection, hepatic resection and aneurysmectomy were excluded. The protocol included information on previous and present cholecystitis, pancreatitis, jaundice, and suspected or proven malignancy; American Society of Anaesthesiologists' (ASA) score; co-morbidity (heart disease, lung disease, liver/renal disease, diabetes with insulin treatment, steroid therapy); diagnostic investigations; and indication for surgery (elective/acute).

### Trial

Eligible patients were considered for inclusion in a randomised controlled trial comparing minilaparotomy and laparoscopic cholecystectomy. Patients were given verbal and written information about the trial and asked if they wished to participate. Exclusion criteria for the trial were: age under 18 years, jaundice, obesity (body mass index >45), pregnancy, cirrhosis of the liver, suspected or proven malignancy, and previous upper gastrointestinal surgery. Recruitment of patients, randomisation, blinding, sample size calculation, control panel and rules for stopping the trial, data collection, and processing have been described in previous reports [7,8]. During the inclusion period, 1705 cholecystectomy patients were identified. Scrutiny of medical records yielded another 14 non-randomised patients. 726 patients were randomised, but two randomised patients were withdrawn: one patient unexpectedly had disseminated malignancy found at laparoscopy and the other patient was randomised to laparoscopic cholecystectomy but was operated with minilaparotomy for unknown reasons. This change was detected too late in data handling to allow collection of necessary information. Analysis was otherwise performed on an "intention-to-treat" basis for both randomised and non-randomised patients. Thus, a converted laparoscopic operation was classified as laparoscopic cholecystectomy and mini-laparotomy cholecystectomy with extended incision (beyond 8 cm) as mini-laparotomy cholecystectomy. 277 patients were excluded from the trial according to protocol (exclusion criteria), 472 patients were excluded by surgeons in charge, and 244 patients, did not want to participate in the trial. Thus, characteristics and outcomes for 993 non-randomised patients and 724 randomised patients could be compared. Approval of the Ethics Committee was obtained.

### Surgery

Laparoscopic cholecystectomy was performed according to routines at the participating units. Mini-laparotomy was defined as cholecystectomy performed through a laparotomy incision less than 8 cm. Trainees were encouraged to participate in surgery under supervision, as is routine practice. Complications were classified according to Clavien et al [9].

### Statistics

Outcome of qualitative variables, for example acute cholecystitis (yes/no) or co-morbidity (no/at least one disease or item) was compared using chi-square analysis. The distribution of quantitative variables (e.g. operating time, hospital stay) was compared using the Kolmogorov-Smirnov non-parametric two-sample test. Mortality within 90 days and from 91 to 365 days of surgery was calculated as the standardised mortality ratio (SMR) i. e. ratio between the observed and expected number of deceased patients, taking into account sex and age of individuals. Mortality after cholecystectomy in a defined population is raised above that of the standard population until 90 days after operation [4] and therefore this postoperative period was used for assessment of postoperative mortality.

Among non-randomised patients, operation time and hospital stay for open cholecystectomy, mini-laparotomy cholecystectomy, and laparoscopic cholecystectomy were compared using multiple regression analysis with open cholecystectomy as a reference. First, the influence of acute cholecystitis, pancreatitis, jaundice, malignancy, co-morbidity, ASA-score, common bile duct exploration, bilio-digestive shunt operation (choledocho-duodenostomy), concomitant operation, and acute operation was tested using univariate analyses. Adjustments were then made for these variables in a multiple regression analysis.

## Results

### Non-randomised versus randomised patients

Table 1 shows the characteristics of trial patients and non-randomised patients. The gender distribution did not differ between the two groups. Trial patients were younger and less likely to have acute operations, complications of gallstone disease, associated diseases, and ASA scores III and IV. Malignancy was identified in 27 of 993 non-randomised patients, 2.7%.

The procedures used for non-randomised patients are shown in Table 2. 52.4% of these patients were treated with conventional open cholecystectomy, 15.5% with mini-laparotomy, and 32.1% with laparoscopic cholecystectomy. The trial protocol did not exclude concomitant operations, but these were more common among non-randomised patients (6.9% vs 2.8%). Operation time, re-operation rate, and re-admissions did not differ between trial patients and non-randomised patients, whereas non-randomised patients had a longer postoperative stay (p < 0.001) (Table 3). As shown in Table 4, the risk of death for non-randomised patients significantly exceeded that of the standard population within 90 days of surgery.

### Operation time and postoperative hospital stay in non-randomised patients

According to multiple regression analysis, operation time for mini-laparotomy cholecystectomy and open cholecystectomy in non-randomised patients did not differ significantly, whereas laparoscopic cholecystectomy took significantly longer (20 minutes) than open cholecystectomy (Table 5). Hospital stay was significantly shorter for both mini-laparotomy cholecystectomy and laparoscopic cholecystectomy compared to open cholecystectomy.

## Discussion

The principal findings of present study are that non-randomised patients were older and more sick than trial patients and more likely to have an acute operation. Of all non-randomised patients 67.9% were operated with open methods, 52.4% with conventional open cholecystectomy, and 15.5% with mini-laparotomy cholecystectomy. In contrast to trial patients, non-randomised patients had a threefold excess death rate compared to the background population during the postoperative period. Multivariate analyses demonstrated that operation time for non-randomised patients did not differ between open cholecystectomy and mini-laparotomy, whereas laparoscopic cholecystectomy took 20 minutes longer. Postoperative hospital stay was shorter for both mini-laparotomy cholecystectomy and laparoscopic cholecystectomy compared to open cholecystectomy.

The strength of the study is the completeness of the data. All cholecystectomies performed during the inclusion period are taken into account. The weaknesses of the study primarily relate to timing. Our study was conducted at a time when laparoscopic cholecystectomy had been in use for five years in Sweden and surgeons' experience with mini-laparotomy was limited. Cholecystectomy was not performed as day-case surgery at the time of the trial, but day-cases have since been introduced both for laparoscopic cholecystectomy and mini-laparotomy at the hospitals participating in the present trial.

Cholecystectomy in elderly and vulnerable patients, who often have complications of gallstone disease, is associated with considerable mortality [5,10]. Therefore, it is an expected observation that our non-randomised patients had a postoperative mortality three times that of the standard population. Whereas 32% of all cholecystectomies performed from 1995 to 1999 in Sweden for acute or chronic gallbladder disease were completed as open operations, the majority of patients over age 70 had an open operation [5]. Under these circumstances, the high percentage of open cholecystectomy among our non-randomised patients is not surprising. Nevertheless, the number of conventional open cholecystectomy compared to mini-laparotomy cholecystectomy is a matter of concern, as randomised controlled trials [11-13] have concluded that the surgical trauma is greater after conventional open cholecystectomy than after mini-laparotomy cholecystectomy.

Our multiple regression analyses indicated that mini-laparotomy was a cost-effective alternative to laparoscopic cholecystectomy among non-randomised patients. This finding agrees with findings in single-blind randomised controlled trials [8,14]. and observational studies [15,16] demonstrating low health care costs for mini-laparotomy cholecystectomy. The effectiveness of an operation can only be assessed on an unselected set of patients and only when surgery has been performed as part of the normal hospital routine. Outcomes after mini-laparotomy cholecystectomy that are comparable to outcomes after laparoscopic cholecystectomy have been reported from units/surgeons where mini-laparotomy is the standard procedure [15-17] or regularly practised [18,19].

## Conclusion

We found that non-randomised patients were more vulnerable than trial patients. Mini-laparotomy should replace conventional open cholecystectomy whenever possible. The versatility of mini-laparotomy has important implications for cost-effectiveness in gallbladder surgery. Low external validity of conventional randomised controlled trials as demonstrated in this report may to a great extent be overcome by expertise based randomised trials [20].

## Competing interests

The author(s) declare that they have no competing interests.

## Authors' contributions

AR, EN, and PC designed the study; AR was responsible for collection of clinical data; and PC, MR, and KB for data analyses. EN drafted the manuscript. All authors read and approved the final manuscript which has been approved by all authors.

## Pre-publication history

The pre-publication history for this paper can be accessed here:



## Figures and Tables

**Table 1:** Characteristics of trial patients and non-randomised patients

		**Women**		**Age, years**		**Acute operation**		**Acute cholecystitis**		**Pancreatitis**		**Jaundice**		**Malignancy**		**Associated diseases**		**ASA III+IV**	
	**n**	**n**	**%**	**Mean**	**SD**	**n**	**%**	**n**	**%**	**n**	**%**	**n**	**%**	**n**	**%**	**n**	**%**	**n**	**%**

**Trial patients**	724	501	69.2	50.6	15.6	106	14.6	72	8.6	0		0		0		154	21.3	28	3.9
**Non-randomised patients**	993	657	66.2	56.5	19.1	516	52.0	405	40.8	53	5.4	140	14.1	27	2.7	336	33.8	99	14.1
		p = 0.18		p < 0.001		p < 0.001		p < 0.001		p < 0.001		p < 0.001		p < 0.001		p < 0.001		p < 0.001	

**Table 2:** Operations on trial patients and non-randomised patients

		**OC**		**LC**		**MC**		**CBDE**		**Shunt**		**Concomitant operation**	
	**n**	**N**	**%**	**n**	**%**	**n**	**%**	**n**	**%**	**n**	**%**	**N**	**%**

**Trial patients**	724	0		362	50.0	362	50.0	39	5.4	0		20	2.8
**Non-randomised patients**	993	520	52.4	319	32.1	154	15.5	201	20.2	15	1.5	69	6.9
								p < 0.001		p < 0.001		P < 0.001	

OC = conventional open cholecystectomy, LC = Laparoscopic cholecystectomy, MC = mini-laparotomy cholecystectomy, CBDE = common bile duct exploration, Shunt = choledochoduodenostomy, Concomitant operation = other operation performed in combination with cholecystectomy.

**Table 3:** Operation time, postoperative stay, and re-operation of trial patients and non-randomised patients

	**N**	**Operation time, min**		**Postoperative stay, days**				**Re-operation**		**Re-admitted within 30 days**	
		**Mean**	**SD**	**Mean**	**Median**	**Percentiles**		**N**	**%**	**N**	**%**

						**25%**	**75%**				

**Trial patients**	724	101	45	2.6	2	1	3	17	2.3	47	6.5
**Non-randomised patients**	993	100	47	4.9	3	2	6	23	2.3	60	6.1
		p = 0.67		P < 0.001				p = 0.92		p = 0.71	

**Table 4:** Standardised mortality ratio (SMR), mean and (95% CI)

		**0 – 90 days**		**91 – 365 days**	
	**N**	**Deaths**	**SMR**	**Deaths**	**SMR**

**Trial patients**	724	1	1.61 (0.02 – 3.46)	3	0.62 (0.13 – 1.78)
**Non-randomised patients**	993	23	3.42 (2.17 – 5.13)	26	1.27 (0.90–1.86)

**Table 5:** Operation time and postoperative hospital stay for non-randomised patients according to multiple regression analysis

	Operation time* min	Postoperative hospital stay** Days
MC – OC	-2.7 p = 0.50	-1.3 p = 0.004
LC – OC	20.2 p < 0.001	-1.2 p = 0.003

MC = minilaparotomy cholecystectomy, LC = laparoscopic cholecystectomy, and OC = conventional open cholecystectomy. Figures indicate adjusted differences between MC or LC and OC.

*Operation time adjusted for common bile duct exploration and concomitant operation.

**Postoperative hospital stay adjusted for pancreatitis, malignant disease, common bile duct exploration, biliodigestive shunt operation, co-morbidity, ASA-score, concomitant operation, and acute operation.

## References

[B1] Charlson ME, Horwitz RI (1984). Applying results of randomised trials to clinical practice: impact of losses before randomisation. BMJ.

[B2] Moher D, Schulz KF, Altman DG (2001). The CONSORT statement: revised recommendations for improving the quality of reports of parallell-group randomised trials. Lancet.

[B3] Livingston EH, Rege RV (2004). A nationwide study of conversion from laparoscopic to open cholecystectomy. Am J Surg.

[B4] McMahon AJ, Fischbacher CM, Frame SH, MacLeod CM (2000). Impact of laparoscopic cholecystectomy: a population-based study. Lancet.

[B5] Nilsson E, Fored M, Granath F, Blomqvist P (2005). Cholecystectomy in Sweden 1987–1999. A nationwide study of mortality and preoperative admissions. Scand J Gastroenterol.

[B6] Majeed AW, Troy G, Nicholl JP, Smythe A, Reed MWR, Stoddard CJ, Peacock J, Johnson AG (1996). Randomised, prospective, single-blind comparison of laparoscopic versus small-incision cholecystectomy. Lancet.

[B7] Ros A, Gustafsson L, Krook H, Nordgren C-E, Thorell A, Wallin G, Nilsson E (2001). Laparoscopic cholecystectomy versus mini-laparotomy cholecystectomy. A prospective, randomised, single-blind study. Ann Surg.

[B8] Nilsson E, Ros A, Rahmqvist M, Bäckman K, Carlsson P (2004). Cholecystectomy: costs and health-related quality of life: a comparison of two techniques. Int J Qual Health Care.

[B9] Clavien P-A, Sanabria JR, Strasberg SM (1992). Proposed classification of complications of surgery with examples of utility in cholecystectomy. Surgery.

[B10] Jørgensen T (2000). Treatment of gallstone patients.

[B11] Assalia A, Kopelman D, Hashmonai M (1997). Emergency minilaparotomy cholecystectomy for acute cholecystitis: prospective randomized trial – implications for the laparoscopic era. World J Surg.

[B12] Assalia A, Schein M, Kopelman D, Hashmonai M (1993). Minicholecystectomy vs conventional cholecystectomy: a prospective randomized trial – implications in the laparoscopic era. World J Surg.

[B13] O'Dwyer PJ, McGregor JR, McDermott EWM, Murphy JJ, O'Higgins NJ (1992). Patient recovery following cholecystectomy through a 6 cm or 15 cm transverse subcostal incision: a prospective randomized clinical trial. Postgrad Med J.

[B14] Calvert NW, Troy GP, Johnson AG (2000). Laparoscopic cholecystectomy: a good buy? A cost comparison with small-incision (mini) cholecystectomy. Eur J Surg.

[B15] Leo J, Filipovic G, Krementsova J, Norblad R, Söderholm R, Nilsson E (2006). Open cholecystectomy for all patients in the era of laparoscopic cholecystectomy: a prospective cohort study. BMC Surgery.

[B16] Seale AK, Ledet WP (1999). Minicholecystectomy: a safe, cost-effective day surgery procedure. Arch Surg.

[B17] Rozsos I, Ferenczy J, Schmitz R (2003). Micro- and minicholecystectomy in the 21st century. Orv Hetil.

[B18] Oyogoa SO, Komenaka K, Ilkhani R, Wise L (2003). Mini-laparotomy cholecystectomy in the era of laparoscopic cholecystectomy: a community-based hospital perspective. Am Surg.

[B19] Syrakos T, Antonitsis P, Zacharakis E, Takis A, Manousari A, Bakogiannis K, Efthimiopolous G, Achoulias I, Trikoupi A, Kiskinis D (2004). Small incision (mini-laparotomy) versus laparoscopic cholecystectomy: a retrospective study in a university hospital. Langenbecks Arch Surg.

[B20] Devereaux PJ, Bhandari M, Clarke M, Montori VM, Cook DJ, Yusuf S, Sackett DL, Cinà CS, Walter SD, Haynes B, Schunemann HJ, Norman GR, Guyatt GH (2005). Need for expertice based randomised clinical trials. BMJ.

